# Oxidative Stress, Inflammation, and Mitochondrial Dysfunction: A Link between Obesity and Atrial Fibrillation

**DOI:** 10.3390/antiox13010117

**Published:** 2024-01-17

**Authors:** Alkora Ioana Balan, Vasile Bogdan Halațiu, Alina Scridon

**Affiliations:** 1 Center for Advanced Medical and Pharmaceutical Research, University of Medicine, Pharmacy, Science and Technology “George Emil Palade” of Târgu Mureș, 540142 Târgu Mureș, Romania; alkora-ioana.balan@umfst.ro; 2 Physiology Department, University of Medicine, Pharmacy, Science and Technology “George Emil Palade” of Târgu Mureș, 540142 Târgu Mureș, Romania; bogdan.halatiu@umfst.ro

**Keywords:** atrial fibrillation, inflammation, mitochondrial dysfunction, obesity, oxidative stress

## Abstract

The adipose tissue has long been thought to represent a passive source of triglycerides and fatty acids. However, extensive data have demonstrated that the adipose tissue is also a major endocrine organ that directly or indirectly affects the physiological functions of almost all cell types. Obesity is recognized as a risk factor for multiple systemic conditions, including metabolic syndrome, type 2 diabetes mellitus, sleep apnea, cardiovascular disorders, and many others. Obesity-related changes in the adipose tissue induce functional and structural changes in cardiac myocytes, promoting a wide range of cardiovascular disorders, including atrial fibrillation (AF). Due to the wealth of epidemiologic data linking AF to obesity, the mechanisms underlying AF occurrence in obese patients are an area of rich ongoing investigation. However, progress has been somewhat slowed by the complex phenotypes of both obesity and AF. The triad inflammation, oxidative stress, and mitochondrial dysfunction are critical for AF pathogenesis in the setting of obesity via multiple structural and functional proarrhythmic changes at the level of the atria. The aim of this paper is to provide a comprehensive view of the close relationship between obesity-induced oxidative stress, inflammation, and mitochondrial dysfunction and the pathogenesis of AF. The clinical implications of these mechanistic insights are also discussed.

## 1. Introduction

Obesity represents an important public health issue, with a significant increase in incidence and prevalence over the past 50 years [1]. It is currently thought to affect more than one billion people around the globe, and its prevalence is continuously on the rise. Moreover, the prevalence of overweight or obese children and adolescents has increased more than four-fold in the last 40 years, representing a major societal concern [2]. The deleterious impact of obesity is not exclusively attributable to its presence. Many of the negative effects of obesity result from obesity-related diseases, including cardiovascular diseases, diabetes mellitus, metabolic syndrome, respiratory disturbances, chronic kidney disease, or fatty liver disease [1].

**Figure 1:**
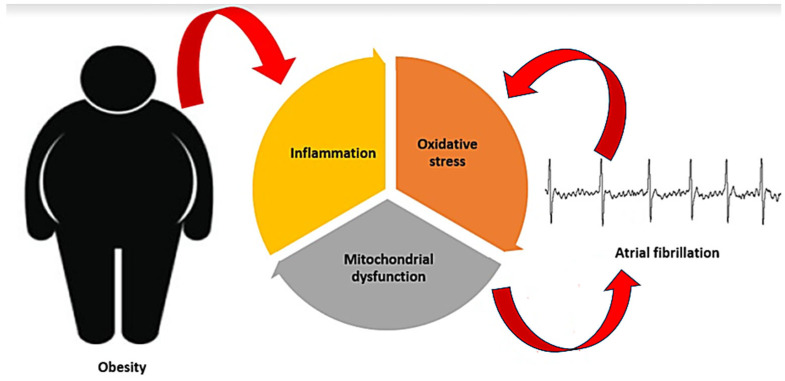
The close relationship between the pathogenesis of atrial fibrillation and obesity-induced oxidative stress, inflammation, and mitochondrial dysfunction.

Among cardiovascular diseases, atrial fibrillation (AF) is the most common sustained cardiac rhythm disorder, affecting approximately 2% of the European population, according to recent data. Even though the prevalence of the disease is estimated at less than 1% in individuals under 49 years of age, it increases to more than 15% in those over 80 years of age [3,4]. A series of cardiac (e.g., coronary heart disease, arterial hypertension, heart failure) and non-cardiac (e.g., diabetes, hyperthyroidism, chronic kidney disease) conditions are known to be associated with the occurrence and maintenance of AF [5]. Extensive evidence has associated obesity with AF [6,7,8], with obese individuals having a more than two-fold higher risk of developing AF compared to non-obese individuals [9]. Clinical conditions frequently present in obese people (e.g., hypertension, diabetes, obstructive sleep apnea) are major risk factors for AF [9]. Obesity-related hemodynamic changes and myocardial ischemia further increase the risk of AF. Additionally, the fat tissue itself has major implications in AF [9,10]. Visceral fat promotes metabolic processes such as chronic systemic inflammation, oxidative stress, and increased insulin resistance, which ultimately damage the atria and increase the risk of AF. Meanwhile, the epicardial fat exerts a direct impact on the atria through mechanical and paracrine mechanisms, as well as interaction with the cardiac ganglionated plexi [9,10]. Obesity affects cardiac structure and function and contributes to cardiac disorders pathogenesis, particularly via the triad of inflammation, oxidative stress, and mitochondrial dysfunction [11]. The same triad can also provide an explanation for the complex pathophysiological link between obesity and AF (Figure 1).

Obesity contributes to atrial fibrillation by affecting cardiac structure and function, particularly via the triad of inflammation, oxidative stress, and mitochondrial dysfunction. In turn, atrial fibrillation promotes inflammation, oxidative stress, and mitochondrial dysfunction, thereby leading to a self-perpetuating cycle.

**Figure 2:**
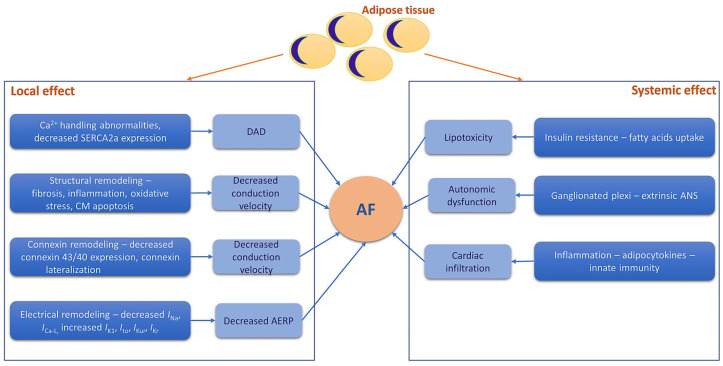
Systemic and local obesity-related factors involved in the pathogenesis of atrial fibrillation. The adipose tissue promotes the occurrence and maintenance of atrial fibrillation both through local effects (Ca^2+^ handling abnormalities, structural, connexin, and electrical remodeling) and systemic effects (metabolic, neurohormonal, and proinflammatory factors). AERP—atrial effective refractory period; AF—atrial fibrillation; ANS—autonomic nervous system; CM—cardiomyocyte; DAD—delayed afterdepolarization; *I*_Ca-L_—L-type Ca^2+^ current; *I*_K1_—inward rectifier K^+^ current; *I*_Kr_—rapid component of the delayed rectifier K^+^ current; *I*_Kur_—ultra-rapid component of the delayed rectifier K^+^ current; *I*_Na_—Na^+^ current; *I*_to_—transient outward K^+^ current; SERCA2a—sarcoplasmic/endoplasmic reticulum Ca^2+^ ATPase 2.

In this paper, we aimed to provide a comprehensive view of the close relationship between obesity-induced oxidative stress, inflammation, and mitochondrial dysfunction and the pathogenesis of AF (Figure 2). The clinical implications of these mechanistic links are also discussed.

## 2. Inflammation in Obesity and Atrial Fibrillation

Both acute and chronic inflammation play an important role in inducing tissue damage. Intracellular pathways are responsible for the production of various inflammatory mediators involved in the occurrence and progression of numerous chronic disorders, including cardiovascular diseases, non-alcoholic fatty liver disease, acute and chronic kidney disease, and lung diseases [12].

A large body of evidence links obesity with chronic inflammation, with the interrelationship between the two being primarily related to the over-expression of proinflammatory cytokines [13,14]. In parallel with its expansion, in obesity, the adipose tissue becomes infiltrated by immune cells, particularly macrophages [13,14,15,16]. This infiltration is a key contributor to the low-grade inflammation that occurs in the adipose tissue. In addition, the adipose tissue per se is an important source of mediators of inflammation, including proinflammatory cytokines and leptin, which directly contribute to inflammation in obese individuals [16,17,18,19]. Obesity also triggers the activation of immune cells, including T cells and macrophages, leading to the release of additional inflammatory mediators [13,14,15,16]. This immune activation is not limited to the adipose tissue but can also affect other organs, contributing to the typical systemic inflammatory syndrome associated with obesity [13,14,15,16]. Increased levels of proinflammatory cytokines, such as tumor necrosis factor-*alpha* (TNF*α*), transforming growth factor-*beta* (TGF-*β*), interleukin-1 *beta* (IL-1*β*), and interleukin-6 (IL-6) were reported in adult obese patients [15]. All these mediators are produced by the macrophages from the adipose tissue via leptin receptor activation [13]. Leptin was found to positively correlate with IL-6 and TNF*α* levels, as well as with clinical parameters of obesity (i.e., body mass index (BMI) and abdominal circumference) [16]. Other studies also showed that central obesity is associated with higher levels of leptin, TNF*α*, and IL-1*β* [17,18]. In a cohort of 740 patients, IL-6 and C-reactive protein (CRP) levels positively correlated with central obesity [19]. In addition to CRP [20], other acute-phase proteins, such as the proinflammatory adipokine serum amyloid A, could also play a critical role in obesity-associated inflammation [21,22] and in obesity-related complications, including AF. The impact of weight loss on the levels of inflammatory markers is still being discussed. In a study conducted by Greco et al., a modest calorie restriction and weight reduction were associated with a significant decrease in leptin levels [23]. Meanwhile, in the study by Rość et al., a decrease in bodyweight by approximately 9% in morbidly obese patients was not associated with a significant decrease in the levels of inflammatory cytokines (IL-6 and TNF*α*) [24]. These results suggest that, at least in morbidly obese patients, only substantial decreases in the amount of adipose tissue, probably leading to BMI normalization, may reflect a significant improvement in obesity-related inflammation [24].

Numerous studies, therefore, point to obesity as a proinflammatory disease. In parallel, studies incriminate obesity as a major risk factor for both the occurrence and maintenance of AF [15,25,26], and inflammation appears to represent one of the key elements of the link between obesity and AF. Inflammation increases the vulnerability to AF through both electrical and structural remodeling [27]. The increased prevalence of AF in inflammatory conditions such as myocarditis, pericarditis, endocarditis, or after cardiac surgery strongly supports the contribution of inflammation to AF development [10,28]. In recent studies performed in patients undergoing coronary artery bypass grafting surgery, preoperative CRP and IL-6 serum levels positively correlated with the occurrence of postoperative AF (Table 1) [29,30]. In the study by Li et al., CRP levels also positively correlated with the risk of AF in the general population [31].

**Figure 3:**
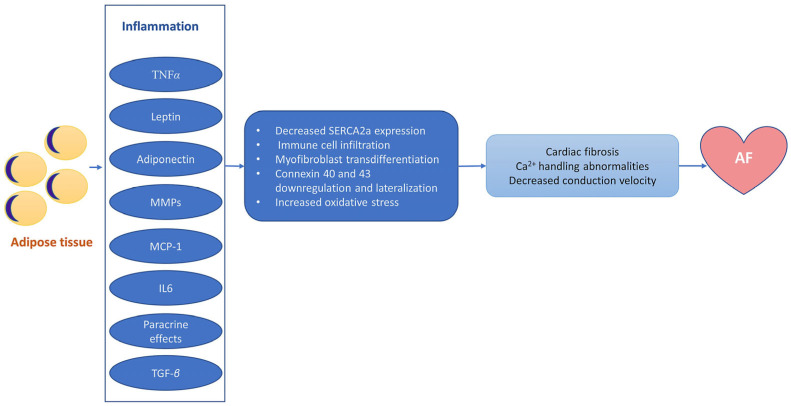
The arrhythmogenic substrate of atrial fibrillation induced by obesity-associated inflammation. A variety of proinflammatory factors originating from the adipose tissue promote the development of proarrhythmic atrial cardiomyopathy involving Ca^2+^ handling abnormalities and structural, connexin, and electrical remodeling. AF—atrial fibrillation; IL-6—interleukin 6; MCP-1—monocyte chemoattractant protein-1; MMPs—matrix metalloproteinases; SERCA2a—sarcoplasmic/endoplasmic reticulum Ca^2+^ ATPase 2; TGF-*β*—transforming growth factor-*beta*; TNF*α*—tumor necrosis factor-*alpha*.

The mechanisms through which obesity-induced inflammation contributes to the onset and maintenance of AF are complex and multifactorial (Figure 3). Chronic inflammation leads to atrial myopathy, characterized by substantial changes in the electrical and structural properties of the atrial tissue. Under the effects of TGF-*β* (via increased *α*-SMA expression), fibroblasts transdifferentiate into myofibroblasts, specialized profibrotic and proinflammatory cells that modify the structure of the extracellular matrix through increased production of collagen [36]. Collagen then contributes to reducing cardiac compliance, interrupting cell-to-cell connections, and decreasing conduction velocity, thus contributing to the typical pathological remodeling observed in AF [37]. In addition, activin A, a member of the TGF-*β* family, has been shown to increase the deposition of fibrotic material in cell culture, supporting the direct role of TGF-*β* in cardiac fibrosis and structural remodeling [38]. In addition, cardiac fibrosis involved in the pathogenesis of AF also appears as a consequence of the upregulation of matrix metalloproteinases and modulation of extracellular matrix degradation via TNF*α* [39]. The adipose tissue is also an important source of IL-2, IL-6, IL-8, and monocyte chemoattractant protein-1. These cytokines contribute to the occurrence and maintenance of AF through cellular (macrophage and neutrophile) infiltration in the myocardium, as well as through inflammation-induced oxidative stress and subsequent fibrosis [37,38,39,40]. All these structural changes induced by inflammation lead to collagen and fibrous tissue deposition within the atria and contribute to progressive loss of atrial compliance and atrial enlargement and stretching, creating the perfect environment for initiation and maintenance of AF [25,27]. Atrial fibrosis also disrupts electrical communication between the adjacent cells and is associated with altered expression and function of ion channels responsible for the generation and conduction of electrical signals within the atria [25,27]. These changes can then lead to alterations in atrial action potential duration and increased spontaneous activity in the atrial cells, increasing the propensity to atrial ectopic activity and the formation of re-entry circuits, thus favoring AF [25,27].

The increased inflammation associated with obesity also leads to electrical changes within the myocardium, contributing to the typical atrial electrical remodeling observed in AF. TNF*α* has been shown to alter Ca^2+^ handling in the cardiomyocytes of the pulmonary veins, probably by decreasing sarcoplasmic reticulum Ca^2+^ ATPase expression, thereby favoring the occurrence of delayed afterdepolarizations and AF [41,42]. Abnormalities in Ca^2+^ handling are also mediated by IL-6, with major implications in atrial arrhythmogenesis [43]. An increase in Na^+^ current density induced by IL-2 via SCN3B overexpression could also contribute to the atrial electrical remodeling observed in AF [44]. In addition, in transgenic mice with cardiac-restricted overexpression of TNF*α*, connexins 40 and 43 were downregulated and lateralized, respectively, with consequences on atrial conduction and atrial arrhythmias [45].

The NOD-like receptor family, pyrin domain containing 3 (NLRP3) inflammasome, is a complex signaling pathway in the immune system that plays a crucial role in the initiation and regulation of inflammation [46]. The NLRP3 inflammasome is an intracellular multiprotein complex with a role in cleaving pro-IL-*β* and pro-interleukin-18 (IL-18) via cysteine protease caspase-1 to generate proinflammatory IL-1*β* and IL-18 [46]. In addition to cytokine maturation, caspase-1 activation triggers pyroptosis, a form of programmed cell death characterized by cell swelling, membrane rupture, and release of cellular contents, which further promotes inflammation [46]. Studies have demonstrated the involvement of the NLRP3 inflammasome pathway in coronary artery disease, acute myocardial infarction, and heart failure, which are major drivers of AF-promoting atrial remodeling [46]. The relationship between NLRP3 activation and AF was demonstrated in an experimental study by Yao and colleagues [47]. In addition, NLRP3 inflammasome activation and regulation seem to have a role in adipose tissue dysfunction and insulin resistance, possibly representing a link between obesity and AF [48].

The relationship between obesity, inflammation, and AF could also be mediated by hypoxia [49]. In AF, an up-regulation of the hypoxia-inducible factor (HIF) pathway and an increased expression of hypoxic and angiogenic markers were observed [49], and the transcription factor HIF-1*α* is upregulated in the adipose tissue in obesity. In obesity, HIF-1*α* contributes to chronic inflammation by promoting the expression of proinflammatory cytokines and recruiting M1 macrophages [50]. The increased expression of HIF-1*α* leads to the activation of a profibrotic transcriptional program, resulting in collagen I, III, IV, and lysyl oxidase synthesis, ultimately causing fibrosis in the adipose tissue [51,52]. HIF-1*α* has also been implicated in the pathophysiology of AF, particularly through structural remodeling, including fibrosis [53]. Ogi et al. observed increased atrial fibrosis in patients with AF that may be secondary to myocardial hypoxia, implicating HIF-1*α* as a key mediator in this process [53]. Moreover, inhibition of HIF-1*α* expression reduced the level of cytokines involved in atrial fibrosis (e.g., TGF-*β*1, MMP-9) and attenuated atrial structural changes [54]. Although a direct relationship between HIF-1*α* and electrical remodeling has not yet been demonstrated, cytokines released due to increased HIF-1*α* activity, such as IL-6 and TNF*α*, have been implicated in proarrhythmic electrical remodeling of the atria [55,56]. This suggests a potential link between HIF-1*α*-mediated inflammation and electrical disturbances contributing to AF. Together, these data suggest that the HIF-1*α* pathway, which is activated in obesity and contributes to chronic inflammation and fibrosis, may also play a role in the pathophysiology of AF. Other transcription factors, including the high-mobility group protein AT-hook 1 (HMGA1), have been identified as participants in the hypoxia-induced inflammatory responses within the adipose tissue [57]. Functioning as an architectural transcription factor, HMGA1 plays a central role in a range of biological processes, including inflammation, tumorigenesis, and metabolism [57,58]. Several studies have shown that HMGA1 physically and/or functionally interacts with nuclear factor-*kappa* B (NF-*k*B) and HIF-1, particularly in the context of hypoxia-associated inflammation, leading to the subsequent release of numerous proinflammatory cytokines [58].

In addition to the considerable amount of data regarding the mechanisms by which inflammation contributes to the occurrence of AF, the inflammation induced by AF per se is also non-negligible. The increased levels of TNF*α* and IL-6 and the activation of the renin–angiotensin–aldosterone and the sympathetic nervous systems commonly associated with AF all contribute to the proinflammatory status observed in the presence of the arrhythmia [59]. Thus, inflammation may also be a result of AF, with a bidirectional relationship between inflammation and AF being present in most patients. This complex relationship may contribute to the development and maintenance of AF and may represent a promising therapeutic target in the treatment of arrhythmia.

The relationship between obesity and AF has been less studied from a genetic point of view. However, variants in genes such as those encoding for cholesteryl transfer protein (CETP), CRP, and G protein-coupled inward rectifier K(+) channel 4 (GIRK4) have been shown to play crucial roles in influencing susceptibility to AF, particularly in the context of obesity. TaqIB of CETP (B2 allele as a protective factor) and CRP 1444 C/T polymorphism may contribute to the susceptibility to AF [60]. In men, these genetic variants were associated with BMI, suggesting a gender-specific genetic influence on the relationship between obesity and AF [60]. Abnormal expression of GIRK4 has also been associated with AF [61]. The connection between *GIRK4* expression and AF was previously highlighted and was shown to be closely related to obesity and metabolic syndrome, suggesting that genetic variations in GIRK4 may contribute to the link between AF and obesity [61].

Several microRNAs (miRNAs) have been identified as common regulators in both obesity and cardiac remodeling. These miRNAs may influence shared pathways related to endothelial dysfunction and fibrosis, providing a possible molecular link between obesity and AF. Although a direct link to AF is not explicitly mentioned, Zou et al. demonstrated that miRNA-410-5p is markedly upregulated in the cardiac tissue of obese rats [62]. The TGF-*β* signaling pathway activation via miRNA-410-5p suggests a potential mechanism for cardiac fibrosis and dysfunction in obesity [62]. As cardiac fibrosis is a common feature in the pathogenesis of AF, miRNA-410-5p could play a role in the relationship between obesity and AF [9]. Altered concentrations of hsa-miR-125a-5p, hsa-miR-342-3p, and hsa-miR-365b-3p in the plasma of obese children with endothelial dysfunction raise the possibility of a connection between these miRNAs and the relationship between AF and obesity [63]. The emerging evidence that endothelial dysfunction is implicated in the promotion and maintenance of atrial arrhythmic substrate and predicts adverse outcomes in AF supports the idea that these miRNAs could play a role in the complex interplay between AF, obesity, and endothelial dysfunction [63,64]. MiR-1-3p and miR-133a-3p were upregulated in extracellular vesicles released from the epicardial adipose tissue [65]. Overexpression of these miRNAs was associated with conduction slowing and reduced *KCNJ2* and *KCNJ12* expression, suggesting that they may act as mediators of epicardial adipose tissue-induced arrhythmogenicity [65]. As the amount of epicardial adipose tissue is directly proportional to the degree of obesity, these findings suggest a link between miR-1-3p and miR-133a-3p and the obesity-AF relationship [65]. In a mouse model of atrial fibrosis induced by a high-fat diet, upregulated miR-205-5p was associated with decreased atrial fibrosis, suggesting that miR-205-5p could represent a therapeutic target for atrial fibrosis-related arrhythmias [66]. The role of these miRNAs and others in the obesity-AF relationship remains to be investigated in future studies.

Inflammation and oxidative stress are interconnected processes, and a complex inter-relationship exists between the two. On the one hand, inflammation stimulates reactive oxygen species (ROS) production. For example, during the inflammatory reaction, immune cells such as macrophages can release ROS to destroy pathogens [67]. On the other hand, inflammation can induce the activity of antioxidant enzymes and oxidative stress defense factors to counteract the harmful effects of ROS [67]. In its turn, oxidative stress also contributes to inflammation [38]. ROS functions as alarm signals for the immune system, initiating inflammation. For example, ROS activates the NF-*κ*B pathway, a key regulator of the inflammatory response [67]. ROS also directly affects lipid and protein inflammatory molecules by causing chemical and functional changes in these molecules [67].

## 3. Oxidative Stress in Obesity and Atrial Fibrillation

The relationship between obesity and oxidative stress is complex and involves chronic inflammation, production of free radicals, impairment of mitochondrial function, antioxidant system imbalance, and other metabolic disorders [10,68,69,70]. All these factors contribute to a bidirectional relationship between obesity and oxidative stress, with obesity increasing oxidative stress and increased oxidative stress contributing to the development of metabolic disorders and diseases associated with obesity [69,70].

Oxidative stress designates an imbalance between ROS production and antioxidants [71]. ROS molecules are produced in the majority of body cells as a result of mitochondrial aerobic metabolism, cytoplasmic enzymatic reactions, or from exogenous oxidant sources (e.g., X-rays, pollutants, cigarette smoking) [72,73], and ROS overproduction increases oxidative stress [74]. At a chemical level, ROS are formed as products of physiological oxygen (peroxide, superoxide, hydroxyl, and singlet oxygen) metabolism [75]. Mitochondria are responsible for the production of over 90% of the superoxide anion by transferring electrons to the oxygen molecule through electron transport chain complexes I/III [73]. In the case of hypoxia, the mitochondrial electron transport chain is disrupted, leading to an incomplete reduction in oxygen molecules and increased generation of ROS [68]. In turn, increased oxidative stress can modify proteins, lipids, and DNA, activating various signaling pathways and leading to cell apoptosis [75].

Even though the white adipose tissue is not considerably rich in mitochondria, normal mitochondrial function is essential in this tissue for the production of energy required for adipocyte differentiation and maturation [73]. Prolonged exposure to ROS of the adipose tissue leads to DNA impairment, resulting in mitochondrial dysfunction and, consecutively, adipogenesis and adipocyte hypertrophy [73]. Oxidative stress, expressed as an overproduction of hydrogen peroxide determined by catalase deficiency, was shown to be involved in obesity through lipo- and adipogenesis [68]. Experimental studies have shown that a high-fat (at least 41% fat) diet in aging C57Bl/6 mice is associated with protein oxidation and increased oxidative stress [76]. In parallel, the activity of antioxidant enzymes glutathioneperoxidase, catalase, and superoxide dismutase is decreased in obese patients [77]. Importantly, weight loss using caloric restriction [78] or bariatric surgery [79] appears to be associated with an improvement in oxidative stress parameters. The use of dietary and supplement antioxidants has also been shown to mitigate oxidative stress and improve obesity and obesity-related conditions [69].

A series of features commonly associated with obesity, such as hyperglycemia, raised levels of plasma lipids and leptin, chronic low-grade inflammation, and altered response to muscle activity, has been associated with the presence of oxidative stress in obesity. Overproduction of nicotinamide adenine dinucleotide and dihydroflavine-adenine dinucleotide as a consequence of an increase in intracellular glucose leads to increased proton gradient across the mitochondrial inner membrane and to superoxide production [80]. Elevated free fatty acid levels promote the production of free oxygen radicals at the mitochondrial level via inhibition of adenine nucleotide translocation [81]. The chronic low-grade inflammation typically associated with obesity also contributes to oxidative stress promotion. Increased levels of TNF*α* and IL-6, commonly encountered in obesity, augment superoxide anion production and nicotinamide adenine dinucleotide phosphate (NADPH) oxidase (NOX) activity [82,83]. The elevation in plasma leptin further amplifies oxidative stress via NOX activation and the production of reactive intermediates [84]. Increased levels of lipid hydroperoxide, isoprostane, and protein carbonyl were observed in animal models following leptin administration [85]. Furthermore, in obese patients, muscle activity during exercise is associated with an abnormal increase in the rate of cellular respiration and oxygen consumption and, consequently, with abnormally high post-exercise lipid hydroperoxide levels [86,87].

In parallel, clinical and experimental studies have demonstrated that oxidative stress contributes to proarrhythmic atrial electrical and structural remodeling through a series of complex mechanisms, thus increasing the susceptibility to AF. Increased levels of ROS (e.g., superoxide and H_2_O_2_) and the ratio of oxidized to reduced glutathione, a marker of oxidative stress, have both been associated with AF [88,89]. Atrial structural remodeling associated with AF seems to be promoted by oxidative damage of myofibrils via hydroxyl and peroxynitrite radicals [90,91]. In addition, mitochondrial DNA damage induced by oxidative stress modulates Ca^2+^ channels and Ca^2+^ handling proteins, leading to Ca^2+^ overload and atrial electrical remodeling [92]. Meanwhile, treatment with antioxidants seems to decrease the risk of postoperative AF. For instance, treatment with vitamin C has been shown to decrease the incidence of AF after cardiac surgery, as well as arrhythmia recurrence after electrical cardioversion of persistent AF [93,94]. Treatment with other antioxidants has also been associated with a lower risk of AF (Table 2). Administration of N-acetyl cysteine was shown to reduce the risk of AF by increasing the density of L-type calcium current [95]. Probucol, xanthine oxidase inhibitors (e.g., allopurinol), selective NOX inhibitors (e.g., apocynin), sodium nitroprusside, and statins, which have potent antioxidant effects, also demonstrated a positive impact on the risk of developing AF [67]. Treatment with antioxidants to reduce ROS and AF risk could also decrease oxidative stress and provide additional benefits in other organs. For instance, n-3 polyunsaturated fatty acids have been shown not only to decrease the incidence of postoperative AF but also to improve musculoskeletal health related to sarcopenia [96]. A healthy lifestyle, with the adoption of a Mediterranean diet, consumption of olive oil, and weight loss, has also been associated with a reduction in oxidative stress and, thus, in the risk of AF [67,97].

Several sources of ROS have been identified in the setting of AF. Among these, NOX, xanthine oxidase, nitric oxide synthase uncoupling, mitochondrial dysfunction, myeloperoxidases, and monoamine oxidases are the most studied sources [67]. The molecular mechanisms underlying the increase in ROS in AF are complex. Although NOX isoform/subunit levels were unchanged, NOX-dependent ROS production and highly upregulated Rac1 expression were observed in a model of pacing-induced AF [112]. Cardiac-specific Rac1 overexpression was also associated with increased prevalence of AF in aged mice, whereas Rac1 downregulation using statins reduced the incidence of angiotensin II-induced AF in endothelial nitric oxide synthase null mice [113,114]. In agreement with animal studies, significant upregulation of Rac1 GTPase and NOX activities has also been observed in patients with AF [114], whereas the use of diphenyleneiodonium and apocynin was shown to inhibit the production of superoxide via the inhibition of flavin-containing oxidases and of p47phox translocation, respectively [115]. Overproduction of NADPH-dependent superoxide was also significantly increased in patients with postoperative AF, whereas atorvastatin treatment attenuated this process, probably by inhibiting Rac1-dependent NOX activation [115,116].

Although the increase in oxidative stress seems to precede the appearance of AF, it could also be a consequence of the arrhythmia. In experimental studies, pacing-induced AF led to NOX-dependent ROS production and upregulation of Rac1 expression, demonstrating that oxidative stress is not only a cause but also a consequence of AF. Moreover, the increase in ROS is also incriminated in the occurrence of consequences of AF, such as thrombosis, inflammation, and even the “AF begets AF” phenomenon [112].

Emerging research has highlighted the pivotal role played by mitochondrial-derived ROS in the intricate landscape of AF [102,117]. Beyond their conventional implications in oxidative stress, mitochondrial ROS are now recognized as crucial components in AF genesis and maintenance. While the focal point often revolves around ROS generated through electron leakage within the mitochondrial respiratory chain, an important role is also played by ROS accumulation resulting from mitochondrial calcium overload.

## 4. Mitochondrial Dysfunction in Obesity and Atrial Fibrillation

The mitochondria’s main function is the production of adenosine triphosphate (ATP) from food substrates, thus playing a central role in energy metabolism [118]. The role of mitochondria extends, however, far beyond the production of energy [118]. During the reactions responsible for ATP production, ROS is also produced at the level of the mitochondria, which represent the main seat of ROS production [118,119]. An exact definition of mitochondrial dysfunction is difficult to formulate. Although it is classically defined as the inability of the mitochondria to generate and sustain sufficient levels of ATP for the cell, metabolic disorders of substrate, Ca^2+^ buffering, mitochondrial DNA mutations, changes in mitochondrial size and morphology, and/or ROS production are also commonly present and can be seen as part of the definition of mitochondrial dysfunction [118,119].

Since excessive nutrient consumption affects mitochondrial function, it is not surprising that obesity is a strong contributor to mitochondrial dysfunction [118]. An increase in free fatty acid concentrations, hyperglycemia, and ROS production, as a consequence of excessive nutrient intake, compromises mitochondrial function at the level of the adipocytes [118]. Further, mitochondrial dysfunction decreases the rate of *β*-oxidation, compromising adipogenesis, fatty acid esterification, lipolysis, and adiponectin production [118]. The relationship between mitochondrial dysfunction and obesity does not seem to be limited, however, to the adipose tissue. A reduction in mitochondrial function and size and in mitochondrial fission, with consequent alteration of mitochondrial dynamics (balance between mitochondrial fusion and fission), were also observed in skeletal muscles in mice with both genetic and diet-induced obesity [120]. As a consequence, reduction in fatty acid oxidation and inhibition of glucose transport occurs in muscle tissues in the presence of obesity [121]. Alterations of mitochondrial function associated with obesity have also been shown to occur in the liver. In rats fed for 14 weeks with a high-fat diet, pathological changes included significant fat deposition, liver steatosis, and a disruption in the hepatic mitochondrial quality control processes, evidenced by increased mitochondrial ROS production, mitochondrial DNA damage, impaired mitochondrial biogenesis, and disrupted mitochondrial fusion [122]. Hepatic mitochondrial fission processes are increased, with consequences on mitochondrial respiratory capacity and protein expression, including peroxisome proliferator-activated receptor-*γ* coactivator-1*α* (PGC-1*α*) [123]. Under normal conditions, only a small amount of the oxygen uptaken by the mitochondria is released in the form of ROS [124]. In the presence of mitochondrial dysfunction, there is a significant increase in the production of ROS, with consequences on mitochondrial and nuclear nucleic acids, membrane lipids and proteins, and the enzymes of the mitochondrial respiratory chain [124]. This entire process is significantly amplified by the aging of the adipose organ, which leads to alterations in adipogenesis, insulin resistance, abnormal adipokine secretion, inflammation, mitochondrial dysfunction, and cellular and tissue senescence [125,126].

At the level of the heart, more than two-thirds of the energy required for sustained contraction and relaxation comes from the oxidation of fatty acids in the mitochondria. The price of this process is the concomitant production of a certain amount of ROS [127]. However, in physiological conditions, this amount is negligible, as it is rapidly removed by antioxidants [127]. In patients with insulin resistance, such as those with obesity, fatty acid oxidation becomes exceedingly important for energy production, the cardiomyocytes shifting away from glucose utilization [127,128]. Consequently, there is an increase in electron leakage and ROS production, ultimately leading to mitochondrial dysfunction [128,129].

Both experimental and clinical studies have intensively studied the issue of mitochondrial dysfunction in AF. In patients with AF, oxidative stress is increased, and damage to the mitochondrial DNA occurs, altering the bioenergetic function of the mitochondria [92,130]. In parallel, oxidative stress and mitochondrial dysfunction have been shown to contribute to the onset and progression of AF. Together, these data indicate the existence of a bidirectional relationship between mitochondrial dysfunction and AF [92,117]. Data suggest, however, that changes in the function of the mitochondria are already present before AF onset and that, once installed, AF accelerates further changes in mitochondrial function [131]. The exact mechanisms through which mitochondrial dysfunction contributes to AF are incompletely understood. Mitochondrial alterations have been shown to contribute to the electro-pathology of the arrhythmia [132]. Mitochondrial functional and structural remodeling appears to be involved in the pathogenesis of AF by increasing energy deficit and metabolic dysregulation in the human and mouse atria [132]. Low ATP levels affect the intracellular ion balance, decrease the efficiency of all energy-dependent enzymatic reactions, and alter myocardial contraction and relaxation, all of which have been involved in the pathogenesis of AF [131]. Increased ROS (especially superoxide anion) generation and apoptotic cascade activation associated with mitochondrial dysfunction also contribute to AF occurrence [132]. Increased O_2_^-^ oxidizes numerous intracellular targets, including the ryanodine receptor 2 of the sarcoplasmic reticulum and the sarcolemmal inward Na^+^ channels, altering cardiomyocyte excitability and intercellular coupling, and thus contributing to maintaining re-entry circuits [133].

Considering the association between mitochondrial dysfunction and AF, it is not surprising that restorers of mitochondrial function have a positive impact on the pathogenesis of AF. Studies have pointed to SS31, a ROS scavenger that improves mitochondrial function by normalizing ATP levels, mitochondrial membrane potential, and mitochondrial morphology, as a promising therapeutic compound in AF [131]. Dipeptidyl peptidase-4 inhibitors, a class of oral antidiabetics, have been shown to improve mitochondrial membrane potential and mitochondrial biogenesis via activation of the PGC-1*α*/NRF1/Tfam signaling pathway and to decrease the duration of pacing-induced AF in a rabbit model of heart failure [134,135]. Sodium-glucose co-transporter 2 inhibitors reduced tachypacing-induced AF susceptibility by approximately 50% in rats with high-fat diet/streptozotocin-induced diabetes mellitus [136]. The mechanisms potentially involved in this effect are suppression of mitochondrial ROS production, preservation of the barrier function of cardiac microvascular endothelial cells via adenosine monophosphate protein kinase activation-induced mitochondrial fission inhibition, as well as restoration of mitochondrial membrane potential and mitochondrial respiratory rate [136,137]. Other therapeutic strategies that could have a positive impact on the pathogenesis of AF by modifying mitochondrial function are presented in Table 3.

## 5. Clinical Implications

Accumulating evidence supports a close relationship between the presence of obesity and AF pathogenesis. Given the continuous rise in the prevalence of obesity in the general population, the burden of obesity-related AF is expected to become increasingly important in the near future. Further research will have to provide new therapeutic options with an impact on the genesis and persistence of AF in obese subjects. Several studies have evaluated the effects of proinflammatory activity, oxidative stress, and mitochondrial dysfunction modulation through weight loss, physical activity, and various drugs in obese patients. Although most of these therapeutic interventions managed to efficiently reduce the inflammatory status, ROS, and mitochondrial dysfunction, more aggressive interventions may be needed to modulate the critical processes involved in AF pathogenesis.

Inflammatory mechanisms are implicated in obesity and are critical contributors to AF occurrence and persistence. Different studies have suggested weight loss as a prevention strategy for AF [8]. Reduction in inflammation in obese patients using direct or indirect/pleiotropic anti-inflammatory agents has not managed to demonstrate a clear benefit in the studies carried out so far, suggesting that multitarget pharmacological interventions along with weight loss may be required and that such strategies may need to be applied at an early stage of obesity. The most appropriate therapeutic strategy aimed at preventing atrial remodeling and AF occurrence in obese people remains to be established. The relative impact of inflammation in different stages of atrial remodeling also remains to be elucidated.

Although the prognostic role of inflammatory markers in AF has been well established, the identification of biomarkers that can be used for the diagnosis and prediction of AF in obese patients would be of interest. To date, a series of such biomarkers have been proposed, but no cut-off points have been established, and, more importantly, none of them is specific to AF. Whether such inflammatory markers have additive value in obese people beyond conventional clinical and echocardiographic risk factors needs further confirmation. In recent years, extensive studies have demonstrated the role of miRNAs in inflammation associated with both AF and obesity [65,66]. However, the full impact of miRNAs in obese patients with AF is still unknown. Further studies should evaluate if there are specific miRNAs present both in obesity and AF and, if there are, evaluate whether these miRNAs can be used for diagnosis and/or prediction or as therapeutic targets in obese patients with AF.

Increased oxidative stress observed in obese patients activates a series of processes (including atrial inflammation, fibrosis, and electrical remodeling) that are, in turn, involved in the occurrence of AF [153,154]. But, these processes, including oxidative stress, are also a consequence of AF. As a result, ROS inhibition in obese subjects may serve as a new therapeutic strategy for breaking the oxidative stress-AF vicious circle. A series of agents with antioxidant properties (e.g., vitamin C alone or in combination with vitamin E or n-acetylcysteine, statins) have been extensively studied in AF, but the results of clinical trials have been rather unconvincing. The answer could come from more specific strategies that specifically inhibit ROS-generating pathways or from siRNAs that target different NOX isoforms.

Considering that the increase in ROS in obese people precedes the appearance of AF, a series of markers of oxidative stress could emerge as biomarkers for the prediction of AF in obese people. The strong correlation that appears to exist between AF initiation and oxidative stress could also be useful for identifying novel biomarkers for AF diagnosis in obese patients. Changes in ROS have also been shown to occur alongside the progression of AF. For example, increased expression of p22phox and NOX2 was observed in patients with postoperative AF. This was not the case in patients with permanent AF [155], suggesting that oxidative stress is a dynamic process and that certain biomarkers could be used to determine different evolutionary stages of AF. Determination of these stages using various biomarkers could have implications in the decision to convert patients to sinus rhythm. However, considering the high degree of variability of oxidative stress, finding a biomarker from this spectrum is a real challenge, at least at this moment.

The contribution of mitochondrial dysfunction to AF and obesity and the molecular mechanisms potentially involved in the obesity-AF relationship have been intensively studied. However, pharmaceutical and nutraceutical compounds directed at mitochondrial dysfunction as a treatment strategy in AF have not been investigated in detail yet. Although many drugs with an impact on mitochondrial dysfunction have been studied both experimentally and clinically, there are still gaps in the knowledge regarding their true impact on the prevention of AF and its clinical outcomes. Further randomized clinical trials could contribute to expanding the indications of certain drugs that are already used in cardiovascular diseases or diabetes for AF prevention in obese people or for prevention of AF-related clinical consequences.

The role of the microtubule network in mitochondrial and cardiac function is well known. Recent studies have shown a key role of microtubule disruption in AF. In cardiomyocytes, overexpression of histone deacetylase 6 (HDAC6), a compound that mediates *α*-tubulin acetylation, responsible for the traffic of metabolites through the cell, results in deacetylation and degradation of microtubules [156]. Experimental and clinical studies have shown that both HDAC6-induced deacetylation and degradation of the microtubule network may underlie mitochondrial dysfunction in AF [156]. However, data regarding the manipulation of the microtubule network in obese patients remain scarce. Elucidating the role of the microtubule network in mitochondrial function may provide further insights into mitochondrial dynamics in obese people and reveal new therapeutic strategies in AF, as suggested by some experimental studies [157]. Biomarkers of mitochondrial dysfunction, such as 8-hydroxy2′-deoxyguanosine, circulating cell-free mitochondrial DNA, and heat shock proteins, could also emerge as promising clinical tools for the prediction and diagnosis of AF in obese people.

## 6. Conclusions

Atrial fibrillation is the most common cardiac arrhythmia and is associated with increased morbidity and mortality. Studies suggest that up to 20% of AF cases can be attributed to being overweight or obese. Although a plethora of obesity-related diseases, including obstructive sleep apnea, diabetes, metabolic syndrome, and dyslipidemia, contribute to the onset and maintenance of AF, obesity per se has a major contribution to AF occurrence, mainly via inflammation, mitochondrial dysfunction, and oxidative stress. These three processes are interdependent, creating a self-perpetuating cycle that contributes to atrial proarrhythmic structural and functional changes, eventually leading to the appearance and maintenance of AF. It is, therefore, not surprising that weight loss is a broad treatment that addresses several central AF mediators, as it is associated in several studies with a decrease in AF load. Meanwhile, a series of anti-inflammatory and/or antioxidant strategies act at the molecular level, counteracting the harmful effects of inflammation, oxidative stress, and mitochondrial dysfunction, and may thus emerge as possible therapeutic agents with a role in AF prophylaxis. Further studies will have to clarify if and when these therapeutic strategies should be used to prevent the occurrence of AF and/or decrease the arrhythmic load.

## Figures and Tables

**Table 1:** Inflammatory markers associated with atrial fibrillation and obesity.

Marker	Study Population	Results	References
IL-6	407 patients with metabolic syndrome, of which 128 patients with AF	Increased levels in patients with AF and metabolic syndrome compared with patients with AF but without metabolic syndrome; positively correlated with left and right atrial volumes and epicardial fat thickness.	[32]
CRP	407 patients with metabolic syndrome, of which 128 with AF	Increased levels in patients with AF and metabolic syndrome vs. patients with AF but without metabolic syndrome; positively correlated with epicardial fat thickness.	[32]
IL-10	CL57/B6 mice divided into high-fat and normal-fat diet groups	Reduced serum levels of IL-10 in high-fat diet-induced obesity.	[33]
MMP-9	105 patients with BMI > 30 kg/m^2^	Significantly higher in patients with obesity and paroxysmal AF vs. patients with obesity without AF; significantly correlated with left atrial volume.	[34]
TGF-*β*	Sheep with and without calorie-dense diet	Increased atrial TGF-*β*, atrial fibrosis, epicardial fat infiltration, and duration of induced AF.	[35]
TNF*α*	407 patients with metabolic syndrome, of which 128 with AF	Increased levels in patients with AF and metabolic syndrome compared with patients with AF, but without metabolic syndrome; positively correlated with epicardial fat thickness.	[32]

AF—atrial fibrillation; CRP—C-reactive protein; IL-6—interleukin-6; MMP-9—matrix metalloproteinase-9; TGF-*β*—transforming growth factor-*β*; TNF*α*—tumor necrosis factor-*alpha.*

**Table 2:** Therapeutic strategies with positive impact on oxidative stress and atrial fibrillation and obesity pathogenesis.

Antioxidant	Disease	Population/ Model	Mechanism	Results	References
Vitamin C	AF	Patients with elective coronary artery bypass grafting	Downregulation of nicotinamide adenine dinucleotide phosphate oxidase	↓ incidence of post-coronary artery bypass grafting AF	[98]
	Obesity	Overweight students	ROS reduction	Attenuation of urinary 8-hydroxy-2′ -deoxyguanosine levels	[99]
Vitamin E	AF	Patients with elective coronary artery bypass grafting	ROS reduction	↓ incidence of post-coronary artery bypass grafting AF	[100]
	Obesity	C57BL/6J mice fed a high-fat diet	Decreases oxidative stress	Increased levels of lipid peroxidation and advanced oxidation protein products	[101]
Statins	AF	Patients undergoing cardiac surgery	Reduces myocardial O_2_ and ONOO^−^	↓ incidence of post-coronary artery bypass grafting AF	[102]
	Obesity	Rats fed a high-fat diet	Reduces renal oxidative stress	Decreased malondialdehyde and glutathione levels; decreased membrane expression of Nox4 and p67^phox^	[103]
n-3 polyunsaturated fatty acids	AF	Patients with coronary artery bypass grafting	Increase electrical stability	↓ incidence of postoperative AF	[104]
	Obesity	C57BL/6 mice fed a high-fat diet	Oxidative stress reduction	Reduced 4-hydroxy-2-nonenal	[105]
N-acetylcysteine	AF	Patients undergoing coronary artery bypass and/or valve surgery	ROS reduction	↓ incidence of post-coronary artery bypass grafting AF	[106]
	Obesity	3T3-L1 and C3H/10 T 1/2-clone 8 (C3H) adipocytes	ROS reduction	Inhibited hydrogen peroxide-induced oxidative stress	[107]
Thiazolidinediones	AF	Rabbits with congestive heart failure	Decrease nicotinamide adenine dinucleotide phosphate; induce antioxidant enzymes such as Cu/Zn superoxide dismutase	Attenuated atrial structural remodeling and inhibited AF promotion	[108]
	Obesity	Obese, hypertensive, type II diabetes rat model	Reduced renal oxidative stress	Reduced nicotinamide adenine dinucleotide phosphate oxidase in kidney tissues	[109]
Probucol	AF	Right atrial pacing AF model	Reduces atrial oxidative stress and increases total antioxidant capacity	Reduces AF promotion and maintenance	[110]
	Obesity	Mice fed a high-fat diet	Reduces oxidative stress	Reduced blood levels of oxidized low-density lipoprotein and malondialdehyde	[111]

↓—decreased; AF—atrial fibrillation; ROS—reactive oxygen species.

**Table 3:** Therapeutic strategies with positive impact on mitochondrial function and atrial fibrillation and obesity pathogenesis.

Medication	Mechanism at the Mitochondrial Level	Effect on AF	Effect on Obesity
Ubiquinone (coenzyme Q10)	Improves mitochondrial function Antioxidant involved in the electron transport from complex I to complex II and from complex II to complex III of the respiratory chain, membrane stabilizer, and cofactor of mitochondrial uncoupling proteins [138]	AF prevention [139]	Reduction in obesity, oxidative stress, and inflammation in metabolic syndrome [140]
Metformin	Preserves mitochondrial function (through AMPK activation), mitochondrial respiration, and mitochondrial biogenesis (probably via upregulation of PGC-1*α*) [141]	Decreased the incidence of AF by 19% [142]	Activated AMPK and improved mitochondrial respiration in obesity [143]
Fibrates	Improve mitochondrial biogenesis via increased PPARGC1A, GFAP, S100B, DCX NRF1, and TFAM genes expression [144]	Decreased AF prevalence [145]	Regulates visceral obesity and inflammation via PPAR*α* activation in obese females [146]
Trimetazidine	Improves ATP synthesis via inhibition of *beta*-oxidation; improves mitochondrial fusion/fission dynamics via normalization of the expression of factors that regulate mitochondrial biogenesis [147]	Prevented tachycardia-induced atrial ultrastructural remodeling, decreased AF inducibility, and shortened AF duration [148]	Successfully mimics exercise to enhance mitochondrial quality control [149]
Ranolazine	Improves mitochondrial function, attenuates oxidative stress, suppresses apoptosis [150]	Attenuated AF [151]	Attenuated obesity-induced non-alcoholic fatty liver disease and increased hepatic pyruvate dehydrogenase activity [152]

AF—atrial fibrillation; AMPK—adenosine monophosphate protein kinase; ATP—adenosine triphosphate; PGC-1*α*—peroxisome proliferator-activated receptor-γ coactivator-1*α.*
